# Lessons learned from catheter ablation of ventricular arrhythmias in patients with a fully magnetically levitated left ventricular assist device

**DOI:** 10.1007/s00392-021-01958-0

**Published:** 2021-10-28

**Authors:** Leonard Bergau, Philipp Sommer, Mustapha El Hamriti, Michel Morshuis, Denise Guckel, René Schramm, Sebastian V. Rojas, Guram Imnadze, Jan F. Gummert, Christian Sohns, Henrik Fox

**Affiliations:** 1 Clinic for Electrophysiology, Herz- und Diabeteszentrum Nordrhein-Westfalen, Ruhr-Universität Bochum, Georgstr.11, 32545 Bad Oeynhausen, Germany; 2 Clinic for Thoracic and Cardiovascular Surgery, Herz- und Diabeteszentrum NRW, Ruhr-Universität Bochum, Bad Oeynhausen, Germany; 3 Heart Failure Department, Herz- und Diabeteszentrum NRW, Ruhr-Universität Bochum, Bad Oeynhausen, Germany

**Keywords:** Left ventricular assist device, Ventricular tachycardia, Catheter ablation, Ventricular storm, Terminal heart failure

## Abstract

**Introduction:**

Data on catheter ablation of ventricular arrhythmias (VA) are scarce in patients with left ventricular assist devices (LVADs) and current evidence predominantly consists of case reports with outdated LVAD. This prospective observational study reports our experience in terms of catheter ablation of VAs in patients with novel 3^rd^ generation LVADs.

**Methods and results:**

Between 2018 and 2020, nine consecutive patients undergoing a total number of ten ablation procedures for VAs were analyzed. The mean duration between LVAD implantation and catheter ablation was 23 ± 16 months. Acute procedural success was achieved in all patients. VA substrates were not related to the LVAD scarring (cannula) site in the majority of patients. All procedures were conducted without any relevant procedure-related complications. In terms of follow-up, only one patient presented with a repeat episode of electrical storm requiring ICD-shocks 16 months after the initial ablation procedure. Four patients suffered of singular VA effectively treated with antitachycardia pacing via their ICD. The remainder were free of any VA relapse (*n* = 4). Two non-procedure-related deaths occurred during follow-up.

**Conclusions:**

Catheter ablation of VAs in patients with 3rd generation LVAD is feasible and leads to satisfying clinical results in terms of freedom from VA recurrence and quality of life. The majority of arrhythmia substrates in these patients are not directly related to the LVAD cannulation site and may represent a progress of heart failure.

**Graphic abstract:**

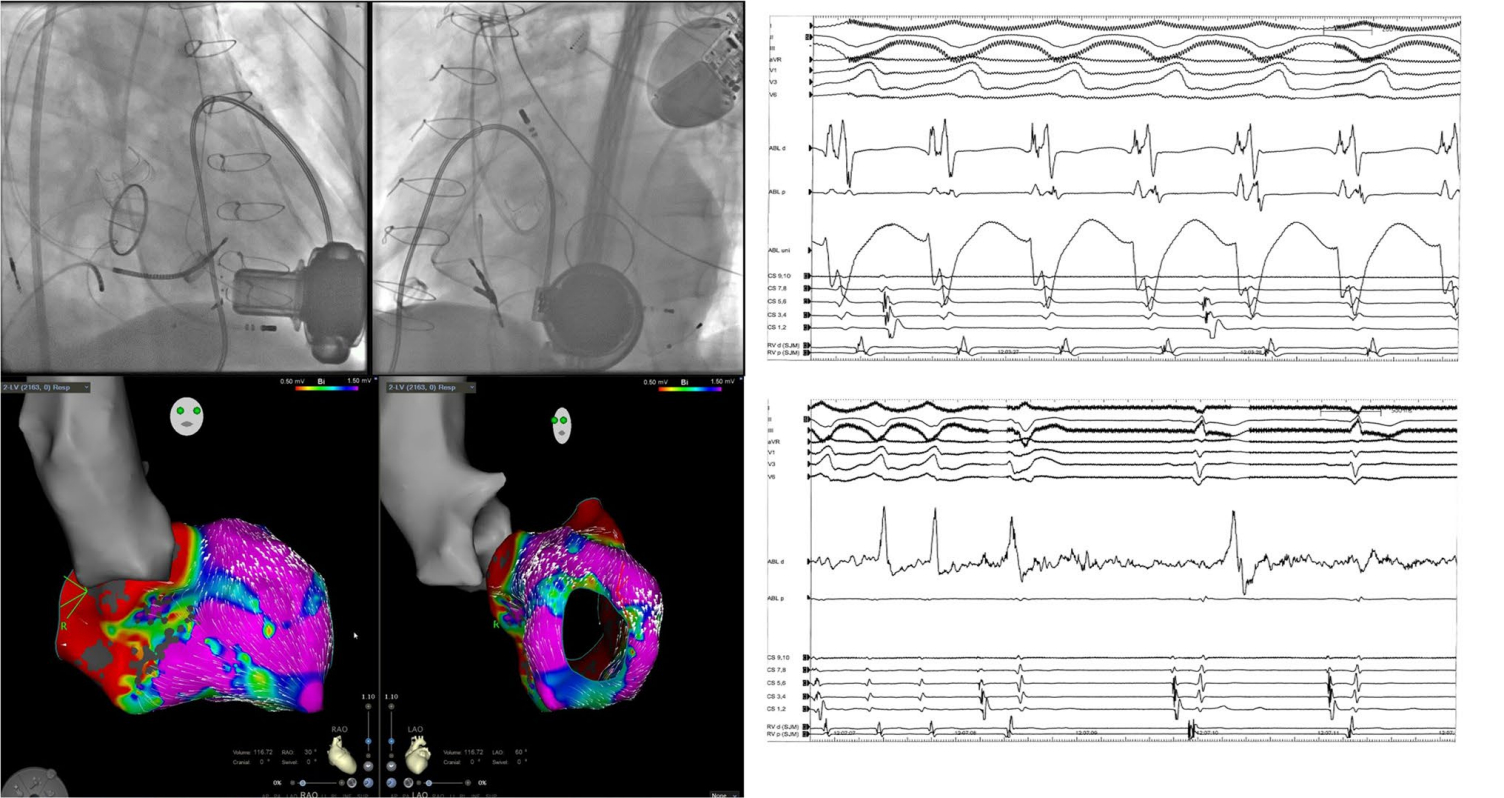

## Introduction

Implantation of left ventricular assist devices (LVAD) as bridge to heart transplant or as destination therapy is increasing in patients with advanced heart failure (HF) [1]. Today, the majority of such patients is implanted with a third generation LVAD with technical reliability and sustained therapy results [2]. These novel devices are small intrapericardially implantable centrifugal-flow pumps [2, 3].

The HeartMate 3 (Abbott, Abbott Park, IL, USA) device is a fully magnetically levitated centrifugal-flow third generation LVAD and this device has shown significant survival benefit over previous axial-flow pump LVADs, e.g., the HeartMate 2 (Abbott, Abbott Park, IL, USA) [2]. The HeartMate 3 device received CE mark in 2015, and FDA approval was granted in 2017. The HeartWare ventricular assist device (HVAD) (Medtronic Inc., Dublin, Ireland) has an impeller suspended in both, magnetic and hydrodynamic forces [3, 4]. The HVAD received CE mark in 2009.

Although the systemic circulation is supported by the LVAD, occurrence of ventricular arrhythmias (VAs) in LVAD patients may have a significant impact on morbidity and mortality [5]. Recurrent VAs are usually treated with initiation or escalation of antiarrhythmic drug therapy, whereas interventional approaches, such as catheter ablation, are rarely performed. There is a lack of evidence and containment of many centers in the context of catheter ablation in LVAD patients. Such procedures may be hindered by unattainability of most VA foci and potential interactions of the mapping and ablation gear with the magnetic field of the LVAD.

Nevertheless, persistent efficacy of antiarrhythmic drug therapy is often limited and recurrent VA episodes including ICD therapy still remain a major issue in LVAD patients with documented increase in mortality for precipitating right heart failure but also mediating quality of life confinements [6, 7].

This study was meant to report on our experience in catheter ablation in 3^rd^ generation LVAD patients.

## Methods

This observational study prospectively included all HeartMate 3 and HVAD patients undergoing VA ablation at our center from October 2018 until December 2020. Indication for ablation was made on individual basis in interdisciplinary agreement between electrophysiology, heart surgery and the heart failure department.

Prior to ablation, all patients received transthoracic echocardiography to rule out LV-thrombus formation and transesophageal echocardiography (TEE) was additionally performed in patients with elevated risk for left atrial (LA) or LA appendage (LAA) thrombus. The ICD/CRT devices were interrogated prior to ablation with deactivation of all VA therapies. Catheter ablation was performed under general anesthesia. Oral anticoagulation was continued aiming for a target-INR between 2.0 and 3.0. During the procedure, heparin was administered to maintain an activated clotting time (ACT) at 300 s. Two diagnostic catheters were introduced via the femoral veins and positioned in the coronary sinus (6 Fr, Webster^®^, Biosense Webster, Inc., Diamond Bar, CA, USA) and the right ventricle (5 Fr, Webster^®^, Biosense Webster, Inc., Diamond Bar, CA, USA). For the antegrade approach, venous access was obtained via right femoral vein and a single transseptal puncture was performed under fluoroscopic guidance using a modified Brockenbrough technique and an 8.5-Fr transseptal sheath (CARTO ViZiGo^®^, Biosense Webster Inc., Diamond Bar, CA, USA or Agilis^®^, St. Jude Medical, Inc., St. Paul, MN, USA). The retrograde access into the LV was obtained via the right femoral artery with insertion of a 8.5-Fr sheath (SL1^®^, St. Jude Medical, Inc., St. Paul, MN, USA), if necessary. All ablation procedures were carried out using a 3D-mapping system (CARTO3^®^, Biosense Webster, Inc., Diamond Bar, CA, USA or Ensite Precision^®^, Abbott Lab., Chicago, IL, USA) and a multipolar mapping catheter (PentaRay^®^, Biosense Webster, Inc., Diamond Bar, CA, USA or HD Grid^®^, Abbott Lab. Chicago, IL, USA). Ultra-high density electranatomical mapping was conducted (aiming for > 1000 points) for the right ventricle (RV) and left ventricle (LV). Low-voltage areas, suggestive for myocardial scar tissue, were defined at bipolar voltage of ≤ 1.5 mV. Radiofrequency current (RFC) was delivered in the power-controlled mode with a maximum power of 40 W, a maximum temperature of 43 °C, and a flow rate of 30 mL/min using an open-irrigated tip-ablation catheter (ThermoCool^®^, Biosense Webster, Inc., Diamond Bar, CA, USA or Flexability^®^, Abbott Lab., Chicago, IL, USA).

Special attention was paid on the 12-lead ECG-filter settings of the EP recording system (Prucka CardioLab^®^, GE Healthcare, Chicago, IL, USA). Best noise reduction was achieved by setting the high pass filter to 0.05 Hz and the low pass filter to 40 Hz.

**Fig. 1:**
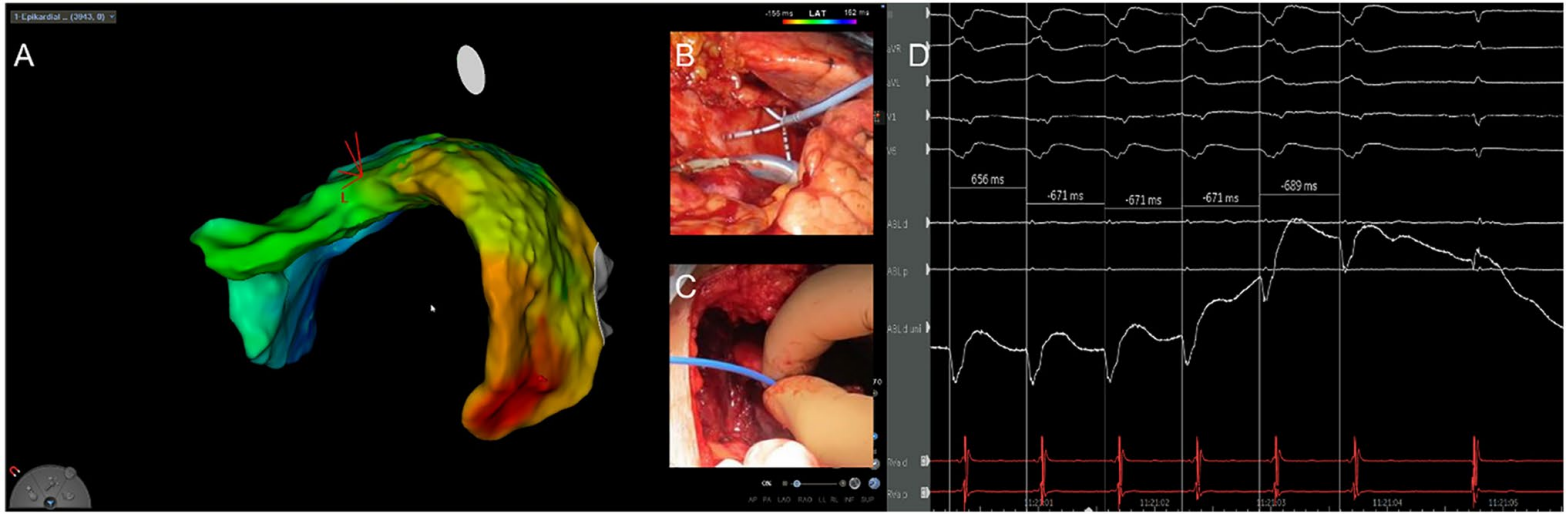
This patient was a 53-year-old male (dilated cardiomyopathy; DCM, LVEF 15%) with previous endocardial ventricular arrhythmia (VA) ablation 2019. In January 2020, a left ventricular assist device (LVAD; HeartWare) was implanted due to recurrent ventricular tachycardia (VT) storm and protracted cardiogenic shock. He was then readmitted to our hospital in September 2020 due to incessant VT storm despite antiarrhythmic therapy. During the VT, the patient was hemodynamically compromised with recurrent low-flow notifications of the LVAD. The VTs were highly suggestive of originating from the epicardium. Taking into account the patients’ refusal to receive a heart transplantation, we decided to perform an open chest epicardial ablation. Following surgical guided epicardial access via left thoracotomy, the epicardium was comprehensively mapped revealing a large scar between the midventricular and posterolateral parts of the left ventricle (LV). After induction of the clinical VT (TCL 420 ms), earliest activation was located at the basolateral site. The VT terminated specifically during ablation and rendered non-inducible during programmed ventricular stimulation (PVS). The patient was then discharged 7 days after the procedure with no other episode during follow-up. **A** Epicardial activation map during ventricular tachycardia (VT). Earliest activation is at the basolateral LV. The epicardium was reached via minithoracotomy. **B** Epicardial HD-mapping using a high-density mapping catheter (PentaRay^©^, Biosense Webster^©^). **C** Epicardial ablation using an irrigated radiofrequency catheter. **D** Specific termination during epicardial ablation at earliest activation site. The patient did not have any recurrence following this hybrid-approach

**Fig. 2:**
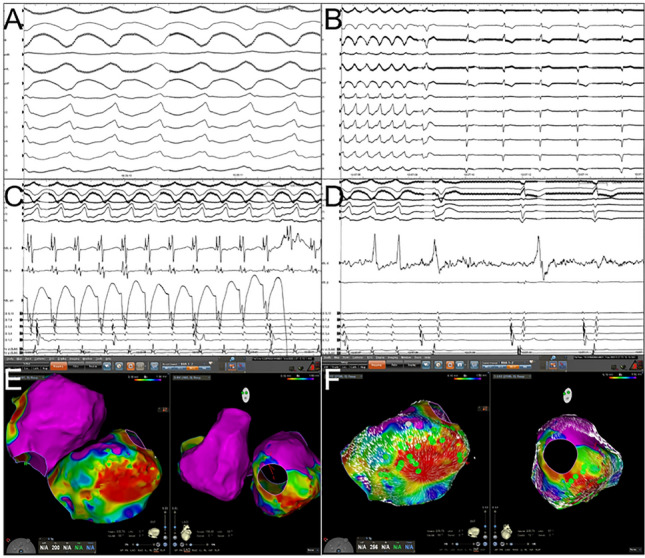
This 62-year-old male patient with ischemic cardiomyopathy and previous inferior infarction (ICM; LVEF 20%) presented in February 2020 due to a ventricular arrhythmia (VA) storm with multiple ATP and shock therapies. Left ventricular assist device (LVAD) therapy was established in 2017 (HeartMate3). Activation mapping showed the earliest activation of the clinical ventricular tachycardia (VT) within an inferior scar. Ablation in this area terminated the VT rendering non-inducible during programmed ventricular stimulation (PVS). After discharge, he suffered one relapse, but after re-establishing a dual antiarrhythmic therapy with Amiodarone and Mexiletine, no more episodes occurred. **A** Sustained ventricular tachycardia during ablation in a patient with HeartMate 3 device. Please note that the ECG-recordings only show slight artifacts. **B** Termination of the VT during ablation at the apicoinferior site. **C** and **D** The intracardiac recordings from the apicoinferior ablation site shows distinct pre-potentials finally leading to specific termination. **E** and **F**: Voltage map (**E**) and coherent map (**F**). Meanwhile the voltage map shows a low voltage scar zone at the apicoinferior site, the coherent map identifies slow conduction at the scar border zones. Green dots: local abnormal ventricular activation (LAVA)

**Fig. 3:**
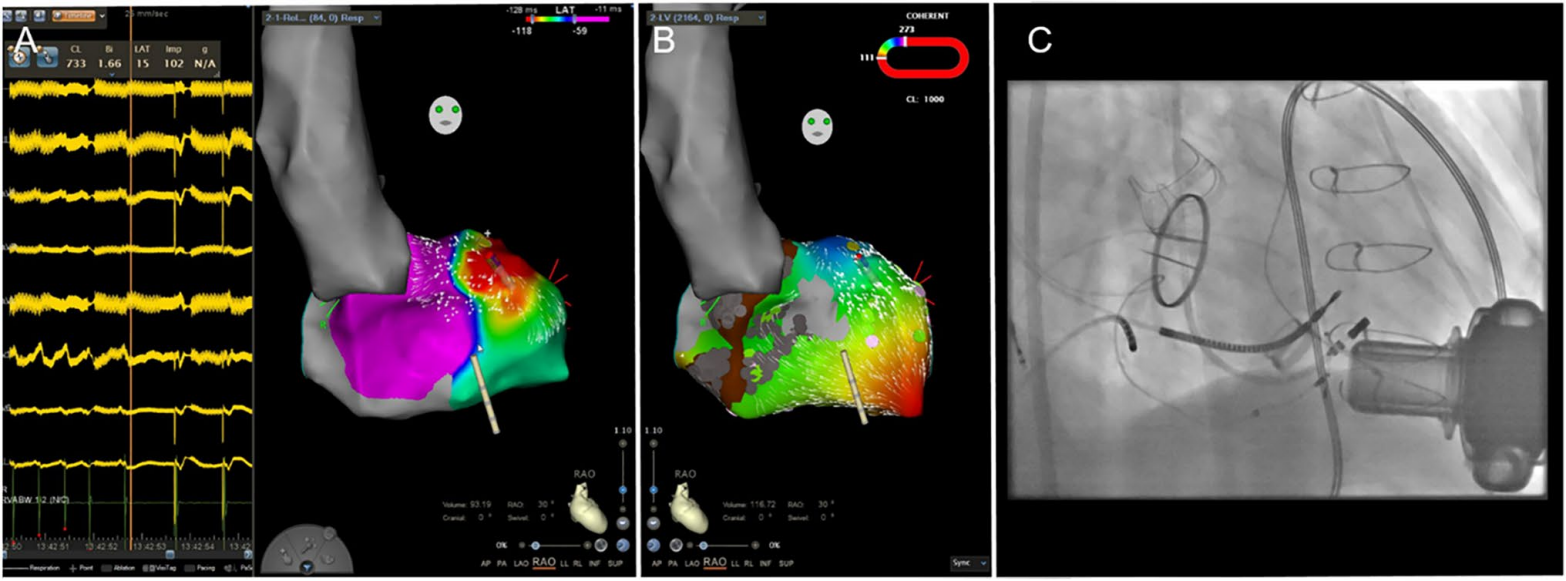
This patient suffered of ischemic cardiomyopathy (ICM; LVEF 25%) with coronary artery bypass graft operation in 2015 in conjunction with mechanical mitral and aortic valve replacement. The mechanical aortic valve was later replaced by a bioprosthetic valve. The left ventricular assist device (LVAD; HeartMate 3) was implanted in 2017. In November 2020, he was admitted to our intensive care unit due to drug refractory ventricular arrhythmia (VA) storm with hemodynamic compromise. We then decided to perform a VA ablation. During the procedure, the left ventricle (LV) was not approachable via transseptal puncture due to the mechanical mitral valve. Further, the aortic valve was permanently closed during the LVAD pump. Hence, access was obtained retrogradely via the aorta by switching off the pump for the passage through the aortic valve. During the procedure and using the 3d-mapping software CARTO 3 (Biosense Webster^©^), termination of the clinical VT (cycle length 280 ms) succeeded at its earliest endocardial activation at the anterior aspect of the left ventricle (LV). Without LVAD, the VT would most likely not have been tolerated. **A** Catheter location at which the VT was specifically terminated. Note the ECG recordings on the left side which shows some noise due the LVAD pump. **B** Coherent map during right ventricular pacing identifies a slow-conduction zone at the spot of successful termination. **C** Fluoroscopy image (RAO 30° view) of the procedure. Please note the mechanical mitral valve which only allowed retrograde access via the bioprosthetic aortic valve. Even in close proximity to the inflow cannula, the catheter was correctly displayed

All ablations were performed according to our local protocol. Briefly, LV and RV high-density voltage mapping was obtained followed by programmed ventricular stimulation when the patient was not in sustained VT at the beginning of the procedure. When VA was sustained, activation map was conducted, and critical isthmus sites were identified using entrainment mapping and novel mapping modules such as the Coherent Mapping algorithm (CARTO 3^®^, Biosense Webster, Inc., Diamond Bar, CA, USA). VT was defined as “cannula dependent” when the isthmus was in close proximity to the cannula insertion site and VA ablation was performed at the critical isthmus site with additional substrate modification as described below. (Figs. 1, 2, 3).

If no sustained VA was inducible, comprehensive substrate ablation was performed targeting all low-voltage areas and abnormal electrograms such as local abnormal ventricular activity (LAVA) and late potentials (LP). After ablation, pericardial effusion was excluded and patients were monitored at our intensive care unit (ICU) for more than 24 h. The ICD/CRT device was reactivated before hospital discharge.

Patients were routinely followed-up in our outpatient clinic for end-stage heart failure therapy at 3, 6 and 12 months. In addition, continuous device (ICD/CRT) interrogations were performed via telemedicine. At each follow-up visit, symptom-specific interviews, physical examination, echocardiography and on-site ICD/CRT interrogations were performed, too. In case of recurrent VA following catheter ablation, all episodes were obtained and analyzed by the treating physicians and redo-ablation was scheduled if applicable.

## Results

### Patient characteristics

Between October 2018 and December 2020, a total of nine LVAD-patients (HeartMate 3 = 5; HeartWare = 4) underwent VA ablation in our hospital. All subjects were male, the mean age was at the time of ablation was 60 ± 8 years, latency of LVAD implantation was a mean of 23 ± 16 months prior to VA ablation. In five cases, the LVAD was implanted with the bridge-to-transplant (BTT) indication and as destination therapy (DT) in four cases. The mean LVEF at the time of ablation was 22 ± 6%. Ischemic cardiomyopathy was present in four patients whereas the remaining five patients suffered of dilated cardiomyopathy. The majority of patients had a history of VAs before LVAD implantation (77%; *n* = 7). Of these, four patients have had initial VA ablation prior to LVAD implantation. At the time of ablation all patients were on ongoing optimal antiarrhythmic drug therapy including Vaughan Williams class III drugs (amiodarone in *n* = 8 and sotalol in *n* = 1). Moreover, three patients even had combined medication of class I, II and III (betablocker, amiodarone and mexiletine). All patients were scheduled for VA ablation due to drug-refractory VA and electrical storm and all patients have had a history of repetitive ICD-therapies prior to hospitalization. In two patients, incessant VA emerged at the time of admission. Of particular note, one patient underwent epicardial thoracotomy guided VA ablation for incessant and therapy-refractory VA as demonstrated in Fig. 1. An ongoing antiarrhythmic drug therapy with amiodarone and betablockers was continued in all cases whereas mexiletine was discontinued if previously described. The one patient with sotalol remained on it after the ablation.

The patients’ baseline characteristics are summarized in Table 1.

**Table 1:** Baseline characteristics of the patients and their respective outcome following the ablation

Patient	Age	Etiology	EF (%)	Type of LVAD	Months between LVAD implant and ablation	Previous VA-ablation	AAD prior ablation	No of ATP/ICD shocks prior ablation	No of ATP/ICD shocks post ablation	Follow-up (days) Status
1	76	DCM	20	HM3	27	No	Amio; Mex; BB	12/2	0	228 Alive
2	47	ICM	25	HVAD	45	No	Amio; BB	11/2	3/0	83 HTx
3	62	ICM	30	HM3	32	Yes	Amio; BB	4/2	6/0	280 Alive
4	64	DCM	30	HM3	22	No	Sotalol	15/3	9/2	254 deceased
5	58	DCM	25	HVAD	40	Yes	Amio; Mex BB	16/1	0	180 HTx
6	53	DCM	15	HVAD	7	Yes	Amio; BB	INC	8/0	153 Alive
7	70	DCM	20	HM3	4	Yes	Amio; Mex BB	20/0	0	22 deceased
8	55	ICM	20	HVAD	37	No	Amio; BB	12/12	0	105 Alive
9	55	ICM	14	HM3	10	No	Amio; BB	INC	0	72 Alive

*AAD* antiarrhythmic drug therapy, *Amio* amiodarone, *BB* betablockers, *EF* ejection fraction, *INC* incessant, *LVAD* left ventricular assist device, *Mex* mexiletine

### Procedural data

All procedures were performed under general anesthesia. The CARTO 3^©^-System in combination with the Pentaray^®^ catheter was utilized in eight cases and the remainder case was performed using the EnSite Precision^®^ System in conjunction with the HD Grid^®^ catheter. Transseptal access was gained in all but one patient that had mechanical mitral valve prosthesis. This patient required retrograde aortic LV approach. An additional retrograde access besides TSP was necessary in five patients. For the epicardial ablation case following LVAD surgery, a left-sided minithoracotomy was conducted to supply pericardial access (Fig. 1). The case is described in detail elsewhere [8], in brief, access was granted through the third to fifth intercostal space followed by blunt dissection to the heart. The pericardium was opened in avoiding damage of the phrenic nerve. The catheters were directly inserted into the epicardial space.

In one patient, we achieved the LV through the permanently closed aortic valve, after a temporal LVAD pump stop to increase the LV pressure towards the aortic valve to gain opening. The pump was reactivated after passage of the ablation catheter into the LV (Fig. 3). Nevertheless, such a maneuver is only possible with farseeing caution and in cases with sufficient remaining LV contractility.

Mean procedure duration (skin-to-skin) was 133 ± 33 min, mean fluoroscopy time was 11.2 ± 6 min and mean fluoroscopy dose showed 1402 ± 1022 (cGy)*cm^2^, respectively. During mapping, a mean of 3040 ± 784 points were acquired. Substrate modification targeting all late-potentials and LAVAS in scar border zones were performed in all cases. In five cases, clinically VA was inducible via programmed ventricular stimulation and additional VA were portrayed in the two patients that had incessant VA. In all cases, sustained VA have been terminated through catheter ablation. After ablation, *n* = 5 patients rendered non-inducible VA during PVS and polymorphic VA was inducible in two patients while in the latter a monomorphic VA other than the clinically documented was inducible but also successfully ablated as described above. Table 2 depicts the evident ablation sites and the scar border zones which were modified during the procedures. Of interest, truly cannula related VA was revealed in solely one patient with dilated cardiomyopathy. This patient already underwent VA ablation prior to LVAD implantation. During the previous ablation procedure, a sustained VA originating from the basolateral LV was successfully treated.

**Table 2:** Procedural details

Patient	Procedure time (min)	Fluoroscopy time (min)	Fluoroscopy dose	Mapping points	Ablation sites
1	103	12.4	687.5	2136	Anterior, septal, inferior
2	139	9.6	994	2643	Ubiquitous
3	120	11.7	3229.8	4479	Inferior, apicoseptal
4	74	6.2	321	3741	Septal RV and LV
5	136	14.7	1714.8	2460	Ubiquitous, LV-summit
6	157	3.2	231	3504	Epicardial, lateral, posterior
7	124	12.4	2390.3	3263	Apical adjacent to cannula site
8	158	24.7	2119	2987	Anterolateral, apical
9	65	6.3	937.8	2152	Septal, anterior

### Arrhythmia recurrence and ablation effectiveness on mortality and LV function

During the entire follow-up period, four patients remained free of any VA recurrence after catheter ablation. The two patients who presented with initial incessant VA developed signs of right heart failure during follow-up and in two patients, slow VA relapse occurred. In contrast to previous events, ATP therapy from the ICD was now effective in these patients. In one patient, VA electrical storm recurred 16 months after ablation.

Two patients received orthotopic heart transplantation 89 and 189 days after VA ablation, (one patient with ATP-sensitive VA relapse and one patient with heart failure progression but without evidence for VA recurrence). Two patients died 22 and 254 days following ablation, both events were not related to VT recurrence based on ICD/CRT interrogation and continuous monitoring. The patient who died 22 days following the ablation suffered of a driveline infection and died due to a septic shock and paralytic ileus. The latter died in hospital due to severe pneumonia. Both deaths were not directly related to the VA-ablation.

## Discussion

### Main finding

This prospective single-center registry reports about feasibility and efficacy of catheter ablation for VA in LVAD patients with both novel HeartMate 3 and HeartWare devices. Our main findings are: VA-ablation in LVAD patients is feasible, effective and safe, even in fully magnetically levitated LVAD. Second, ablation results in terms of freedom from VA recurrence is satisfactory and mid- to long-term results are comparable to VA ablation in non-LVAD patients. Third, the majority of VA foci were not related to LVAD inflow-cannula sites, and our study dismisses previous hypotheses that LVAD implantation would induce VA. Fourth, all VA foci have been reached and targeted during ablation procedures without comprise of LVAD function or hemodynamic interference. On the contrary, the LVAD might even have beneficial effects on the success of VA ablation by maintaining the hemodynamics even in patients with fast VT facilitating the identification of critical isthmuses and re-entry mechanisms. None of the VA ablation procedure was associated with serious adverse events and VA ablation procedures did not negatively impact morbidity or mortality in these distinct patients.

### Feasibility and safety of catheter ablation in patients with LVAD

Current literature hypothesized potential interference between LVAD and electroanatomical mapping systems (EAM) [9] addressing the issue that VA ablation procedures were considered not possible in these LVAD patients. Our study used the magnet-based CARTO 3 (Biosense Webster, Irvine, CA, USA) or the magnet-activated function of the Ensite Precision System (Abbott, Abbott, Park, IL, USA) for all ablation procedures. These mapping systems establish a low-energy magnetic field around the patients’ chest to detect catheter positions in real-time and to allow catheter navigation during ablation. With no changes or adjustments made to LVAD programming during the ablation procedures, we found no significant interferences between LVAD and the mapping system that would have refrain procedure execution. Nevertheless, diligent care is necessary, because catheter tip visualization can be provocative in close proximity to the inflow cannula of the LVAD, but with negligible effect on the procedural efficacy. Our study demonstrated that catheter navigation as well as ablation can be performed safe and effective using a pure fluoroscopic-guided approach in the area around the inflow cannula. Furthermore, no catheter entrapment or procedural related adverse event occurred during the procedures, even when using multipolar mapping catheter.

Due to the heterogeneous and complex substrate in patients with VA, epicardial access is often necessary for complete elimination of tachycardia circuits. However, the preferred method via subxiphoid puncture in patients with previous surgery if often not possible. In this context, Zhang et al. [10] reported on the feasibility of epicardial access using left sided mini-thoracotomy in patients with previous surgery or epicardial ablation procedures. In our study, we report on the feasibility of surgical assisted epicardial mapping and ablation in a patient carrying a LVAD extending the evidence of this approach to this special patient group.

Our study not only shows feasibility of VA ablation in LVAD patients, but also demonstrates effective and safe ablation results with VA termination and additional substrate modification demonstrating freedom from VA recurrence in nearly half the patients. In the remainder with VA relapse, ATP of implanted cardiac devices was more effective compared to before ablation and most ATP stimulation effectively terminated VA episodes. ICD shocks or electrical storm recrudescence became very rare findings after ablation, even in those patients that had incessant VA before ablation.

Another important finding of our study is the observation, that VA substrates in patients with modern LVAD are not unduly associated to the LVAD inflow cannula site [11, 12]. In this context, we can conclude and disprove the hypothesis that LVAD implantation implies relevant risks to induce VA. However, patients qualifying for LVAD implantation suffer from end-stage heart failure and that might potentially explain why these patients are at highest risk of developing VA. In addition, VA occurrence has clearly been shown to drive mortality in end-stage heart failure [13]. Our study cohort represents typical heart failure patients with a history of documented recurrent or even incessant VA episodes prior to LVAD implantation and the modified hemodynamics following LVAD implantation did not augment the presence of VA in this study population.

This is the first prospective observational analysis of 3rd generation LVAD patients undergoing VA ablation and our data indicate convincing procedural safety and effectiveness. However, we feel that such interventions should be performed in a multi-disciplinary setting comprising of electrophysiologists, cardiac surgeons and cardiac anesthetists, not only to provide the option for surgical exposure of epicardial sites, but also to hold all bailout options available, such as extracorporeal circulation, emergency heart surgery, etc.

### Effect on mortality

In a recent meta-analysis including 18 studies with a total number of 110 patients, Anderson et al. reported high acute success rates for VA ablation in LVAD patients (90%) and a significant reduction of VA-related ICD shocks following ablation [14]. With a pooled mortality of 48.1% and a mortality rate of 63.5 per 100 person-years, mortality still remained high in these advanced diseased, end-stage heart failure patients, of whom many await heart transplantation or finally receive palliative care. In this context, additional previous data suggest that effective VA ablation may beneficially impact mortality in selected patients [15, 16].

However, VA recurrence indicates a high risk of premature death in LVAD patients [5, 11, 13, 15], and whether effective VA-ablation has the potential to reduce mortality in these patients has to be investigated in large studies in the future.

Notably, frequent comorbidities in end-stage heart failure patients additionally contribute to increased risk of premature death [17, 18] and comorbidities can make particular heart failure treatments impossible, such as medications in the context of kidney failure [19]. VA ablation appears conductible in most cases, although vascular diseases can account for additional challenge. During study follow-up, two LVAD patients died for non-cardiovascular reasons and both events were not related to catheter ablation or VA recurrence. Reflecting the high risk of premature death in end stage HF and LVAD patients, impact of mortality through VA recurrence is not the only focus to reduce mortality in these patients. Moreover, VA ablation should not only aim to reduce mortality, but has also an indication to reduce VA burden and to improve HF symptoms and QoL in these patients. In summary, no study has shown beneficial prognostic efficacy of VA ablation in LVAD patients to date and large, randomized controlled clinical studies are necessary to address this important issue.

### Troubleshooting and complications

There are reasonable concerns about VA ablation in patients with fully magnetic levitated HeartMate 3 LVAD, because irreconcilable interferences with the 3D mapping system are feared and have previously been reported. Although all ablation procedures were straightforward and highly effective, minor interference and signal disturbance were observed, such as a noisy body surface ECG. Moreover, interference signal affected in some cases the automated template matching algorithm of the mapping software leading to invalid results. Furthermore, the ablation catheter was not correctly displayed when maneuvered in direct proximity to the inflow cannula site. Of note, catheter movement was always possible and safe in this certain area when guided by fluoroscopy and with distinctive precaution and knowledge about signal interference. Of note, all ablation procedures were carried out without any relevant procedure-related complications.

### Limitations

The study only includes nine patients, so the results of the study, especially the patient-specific outcomes, can hardly be transferred to the entire LVAD collective. However, we observed a reduction of ventricular arrhythmias in all patients. Prospective randomized studies in large collectives are necessary to assess the clinical benefit of VA ablation in these patients.

## Conclusion

This is the first prospective study on the feasibility, efficacy and safety of VA ablation in LVAD patients with novel fully magnetically levitated devices. We show effective arrhythmia treatment through catheter ablation using conventional ablation approaches in highly specialized centers for both, end stage HF and complex arrhythmias. Catheter ablation in LVAD patients should be performed in patients presenting with disperse arrhythmia mechanisms and hemodynamic compromise despite LVAD therapy. Arrhythmia origin and mechanism were not related to the LVAD itself in the majority of patients. VA ablation in LVAD patients should not only focus on mortality but can also improve HF symptoms and QoL.
